# Mindfulness-Based Therapies in the Treatment of Functional Gastrointestinal Disorders: A Meta-Analysis

**DOI:** 10.1155/2014/140724

**Published:** 2014-09-11

**Authors:** Monique Aucoin, Marie-Jasmine Lalonde-Parsi, Kieran Cooley

**Affiliations:** Canadian College of Naturopathic Medicine, 1255 Sheppard Ave East, Toronto, ON, Canada M2K 1E2

## Abstract

*Background*. Functional gastrointestinal disorders are highly prevalent and standard treatments are often unsatisfactory. Mindfulness-based therapy has shown benefit in conditions including chronic pain, mood, and somatization disorders. *Objectives*. To assess the quality and effectiveness reported in existing literature, we conducted a meta-analysis of mindfulness-based therapy in functional gastrointestinal disorders. *Methods*. Pubmed, EBSCO, and Cochrane databases were searched from inception to May 2014. Study inclusion criteria included randomized, controlled studies of adults using mindfulness-based therapy in the treatment of functional gastrointestinal disorders. Study quality was evaluated using the Cochrane risk of bias. Effect sizes were calculated and pooled to achieve a summary effect for the intervention on symptom severity and quality of life. *Results*. Of 119 records, eight articles, describing seven studies, met inclusion criteria. In six studies, significant improvements were achieved or maintained at the end of intervention or follow-up time points. The studies had an unclear or high risk of bias. Pooled effects were statistically significant for IBS severity (0.59, 95% CI 0.33 to 0.86) and quality of life (0.56, 95% CI 0.47 to 0.79). *Conclusion*. Studies suggest that mindfulness based interventions may provide benefit in functional gastrointestinal disorders; however, substantial improvements in methodological quality and reporting are needed.

## 1. Introduction

Functional gastrointestinal disorders (FGIDs) have a high prevalence, a significant impact on patients' wellbeing and are costly to the health care system [1]. Patients with these disorders report a marked impact on quality of life and an average of 30 sick days per year per person, constituting a substantial health care burden [2].

The pathophysiology underlying FGIDs is unclear as they lack any discernable organic or structural pathology. Current knowledge suggests the involvement of factors such as abnormal gut motor function, increased visceral perception, abnormalities in central pain processing, and disruption of the gut microbiota as well as genetic and psychological factors [1]. Psychiatric disorders are frequent comorbidities in patients with FGIDs and recent prospective study evidence suggests that the relationship is bidirectional [1].

Of the FGIDs, the most common is irritable bowel syndrome (IBS), affecting 7–10% of the population worldwide. It is characterized by recurring abdominal pain or discomfort and diarrhea or constipation [1].

Standard treatment for IBS is targeted at symptom control through the use of laxatives, antidiarrheal agents, antispasmodics, and antidepressant medications. Studies report that less than 50% of patients with IBS are satisfied with the standard medical treatment and many turn to alternatives. Studies of complementary and alternative medicine use in IBS populations have reported rates of 21–51% [2].

Treatment and burden of other FGIDs such as functional abdominal pain, vomiting, and dyspepsia are less well understood, although there is considerable categorical overlap with IBS. Similarly to IBS, other FGIDs are associated with high rates of complementary and alternative medicine usage. Pharmacological treatments for other FGIDs, aimed at targeting receptors with enteric and central nervous system effects, are similarly in the early stages of development [3–5].

Because of the significant involvement of emotional, cognitive, and neurological factors in IBS, a number of studies have investigated psychological interventions including cognitive behavioural therapy (CBT), hypnotherapy, and relaxation exercises. An early review suggested that all of these interventions have shown benefit [2].

A more recent addition to this list of interventions is mindfulness-based therapy (MBT), a form of psychotherapeutic treatment which uses meditation practices to assist patients in the cultivation of nonjudgemental awareness of the present moment. This involves monitoring of cognition, emotion, perception, and sensations and the development of nonreactivity to difficult or negative aspects of these experiences [6]. The use of mindfulness as a therapeutic tool began in the late 1970s with the development of the mindfulness-based stress reduction (MBSR) program as a treatment for chronic pain [7]. The MBSR program has been combined with CBT in the development of mindfulness-based cognitive therapy (MBCT). It was developed for the prevention of major depressive disorder relapse [7], however evidence to support its use in anxiety and active depression continues to emerge [8]. The programs typically consist of 8 weekly 2.5 hour group sessions involving various forms of meditation, group discussion, and other exercises; one day of meditation retreat and approximately one hour of daily home practice [9].

In addition to the treatment of mental health concerns, there is an expanding body of research supporting the use of mindfulness-based interventions for stress, pain, and somatization disorders such as fibromyalgia and chronic fatigue syndrome [7].

A review article exploring the neural mechanisms of mindfulness and meditation found significant structural and functional changes within the brain both during, and resulting from, mindful states and practices [10]. Based on patterns of cortical thickening, meditation is associated with structural changes in brain regions related to sensory, cognitive, and emotional processing [11].

Because of the significant involvement of emotional factors in IBS, it was initially suspected that the benefit of psychological interventions was achieved through improvement of comorbid psychological distress [6]. A recent study utilized a number of assessment tools to explore some hypothesized mechanisms for the benefit exerted by MBT on IBS. The results of their analysis revealed that several cognitive processes are involved. MBT led to a decrease in reactivity to thoughts, emotions and physical sensations which led to a decrease in visceral sensitivity. The decreased visceral sensitivity was related to a decrease in IBS symptom severity and an improvement in quality of life. Additionally, nonreactivity was associated with a decrease in pain catastrophizing which predicts improvement in quality of life and increased reinterpretation of pain sensations predicted reductions in IBS severity [6].

Previous reviews studying the use of MBT in FGIDs have combined it with other psychotherapeutic interventions or with other disorders [7, 12]. A recent systematic review and meta-analysis investigated the use of mindfulness-based therapy in the treatment of somatization disorders including fibromyalgia, chronic fatigue, and IBS [7]. In the time since this review was completed, additional clinical trials have been published. The review examined efficacy outcomes at the end of treatment only and did not discuss risk of bias or other elements describing the quality of reporting of the studies. A synthesis which includes these components is essential to provide context to the findings as well as provide guidance for future research.

This review will discuss the effectiveness of mindfulness therapy at improving symptom severity and quality of life measures in patients diagnosed with FGIDs compared to waitlist or active control groups. The review will explore the effectiveness at the end of the intervention as well as after a follow-up period. Additionally, the quality of the studies will be assessed to describe the current state of reporting and study bias in the existing literature.

## 2. Methods

The PRISMA statement was used to guide the conduct and reporting of this meta-analysis [13].

### 2.1. Systematic Literature Searches

Systematic literature searches were performed using the Pubmed, EBSCO, and Cochrane databases. The following search terms were used: mindfulness, MBCT, MBSR, mindfulness-based cognitive therapy, mindfulness-based stress reduction, mindful, functional gastrointestinal, functional bowel, colonic disease functional, colonic disease, functional abdominal pain, recurrent abdominal pain, abdominal pain, IBS, irritable bowel, spastic colon, irritable colon, constipation, diarrhea, bloating, distention, gastroesophageal reflux, GERD, dysphagia, and functional dyspepsia. Studies in any stage of publication from database inception onward in English were considered. The purpose of this strategy was to be inclusive of the existing literature and noting that previous reviews did not identify a large base of non-English publications. The last date searched was May 29, 2014.

The search results were combined and duplicates were removed. A screen of article titles and abstracts was performed to identify clinical trials that utilized mindfulness-based interventions for the treatment of FGIDs. After reviewing the full-text articles, those with control groups, randomization, and an adult population with FGID symptoms were included.

### 2.2. Data Collection

Data was extracted by one reviewer. Data for the following study variables was extracted: study size and percent female participants, participant diagnosis, intervention and duration, control, follow-up, symptom severity at the end of the intervention and at follow-up, and quality of life assessment at the end of the intervention and at follow-up. The principle summary outcome measures for synthesis were the changes in symptoms severity between baseline, end-of-intervention, and follow-up. Corresponding authors of included studies were contacted regarding missing or unclear data, though notably this did not result in any additional information beyond what was originally published. Two attempts to contact authors via email were made before ceasing attempts at correspondence.

### 2.3. Data Analysis

Effect sizes (Cohen's *d*) were calculated for relevant validated outcome measures (effect on IBS severity at end of intervention, effect at postintervention follow-up, and quality of life) from individual studies using reported mean, standard deviation and group size. A random effects model (DerSimonian-Laird (DL)) was assumed to account for the small number of studies with pool-able data (*n* = 5-6), small sample sizes, and high degree of variance within the studies. Studies were weighted based on sample size in order to generate a pooled point estimate and 95% confidence interval for effect size. Heterogeneity was assessed using the *I* ^2^ statistic; Cochran *Q* is reported as an inference of combinability of studies. Kendall's tau and Egger's test will be reported to assess for power and risk of bias affecting the cumulative result. Statistical analysis and figure generation (funnel and forest plots) were accomplished using StatsDirect (version 3.0.119) software.

### 2.4. Quality Analysis

Assessment of study quality was conducted using the Cochrane Risk of Bias [22] and the CONSORT checklist for reporting trials of nonpharmacologic treatments [23]. Assessment was completed by two reviewers independently and any discrepancies were discussed until a consensus was reached.

## 3. Results

### 3.1. Literature Search

**Figure 1:**
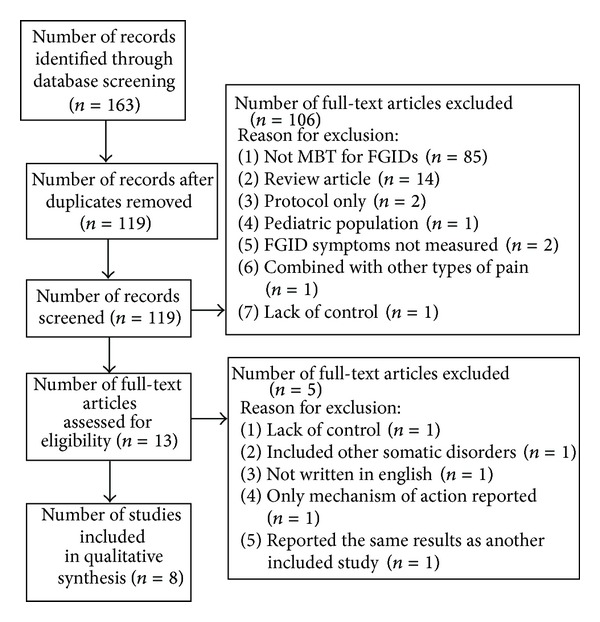
PRISMA flow chart showing number of screened, included, and excluded studies.

The literature search yielded 119 unique records (Figure 1). After these records were screened based on title and abstract, 106 studies were excluded. The reasons included the following: did not assess the use of mindfulness in FGIDs (85), review articles (14), protocol only (2), uncontrolled design (1), pediatric population (1), other types of pain included (1), outcomes limited to cost effectiveness (1), and outcomes limited to psychological symptoms (1). Of the 13 full-text articles assessed for eligibility, eight articles reporting the results of seven randomized controlled trials met the criteria for inclusion in this analysis. The reasons for exclusion were a lack of adequate control (1), combination with other somatic disorders (1), not written in English (1), only mechanism of action outcomes reported (1), and reporting the same results as another included study (1).

### 3.2. Efficacy—End of Intervention

Of the seven studies included in this review, five (71.4%) reported significant improvements in IBS symptom severity at the end of the intervention compared to waitlist or comparison intervention (Table 1). One study did not report end-of-intervention results [24]. One study, which included patients with inflammatory bowel disease (IBD) who were in remission and experiencing IBS-like symptoms, showed a nonsignificant trend towards improvement compared to waitlist control. These patients represented a subgroup analysis within the study and, thus, had a small sample size [14].

### 3.3. Efficacy—Follow-Up

Data from a follow-up time point was reported in all eight publications. These follow-up periods ranged from two to 18 months after the end of the intervention. The study of IBD patients continued to show a trend towards improvement that did not reach significance [14]. The study that only reported data from the follow-up assessment showed significant improvement [21]. The other six studies reported that participants maintained improvement in the severity of their IBS symptoms. Among these, one showed a nonsignificant trend towards further improvement [19]. One study that showed maintenance of improvement showed improvement in the control group resulting in a loss of statistical significance [20]. During the follow-up period the participants did not receive further treatment with mindfulness-based therapy; however the programs taught participants skills and exercises which they were encouraged to continue using. Two studies assessed for the use of additional treatments during the follow-up period and found no significant difference in the outcomes reported by those who had sought additional treatment and those who had not [17, 18].

### 3.4. Efficacy—Quality of Life

Five studies utilized the irritable bowel syndrome quality of life instrument (IBS-QOL) as a secondary outcome and of these, 80.0% (*n* = 4) reported a significant improvement at end-of-intervention. Between the end-of-intervention and the follow-up assessment, significant further improvement was seen in two of these studies while the other two studies showed maintenance of improvement. One study demonstrated a significant improvement in IBS-QOL in both the intervention group and the wait list control group that was maintained at follow-up [20]. The study reporting long-term follow-up data only showed maintenance of QOL improvement.

The study that enrolled IBD patients used an objective biomarker for the assessment of intestinal inflammation [14]; however none of the other studies used objective tests for the assessment of FGID symptoms as primary or secondary outcome measures. All of the assessment tools relied on validated patient/self-report outcome measures.

Two studies [18, 19] used a linear mixed-effects model to observe the difference in rates of change between the MBT and control intervention over time amid significant interaction effects between group and time were seen (*P* < 001).

### 3.5. Quality Assessment

Quality assessment of the studies included in the review revealed strengths as well as weaknesses and opportunities for the introduction of bias. The Cochrane risk of bias assessment showed overall unclear or high risk of bias for the included studies (Table 2).

The most significant contributor to risk of bias was a lack of blinding of participants, facilitators, and outcome assessment. In three studies, the mindfulness intervention was compared with a support group or another psychological intervention and the participants were not aware of their allocation in the study; however, the remaining studies used a waitlist control or treatment-as-usual comparison and in these cases, the participants were aware that they were receiving the intervention being tested. In all studies, personnel who were administering the therapy were not blinded, although this is acknowledged as an inherent challenge in psychological interventions.

Another area that presented a risk of bias is incomplete outcome data. In many studies the rate of withdrawal was the same in the intervention and control groups and intention to treat analyses were utilized; however, in many cases the dropout rates were large, ranging from 10 to 44%. One study failed to report outcome measures at the end of the intervention and only reported data from the follow-up assessment. Two studies failed to describe their funding source. Some studies lacked clarity in their description of random sequence generation (*n* = 1) and allocation concealment (*n* = 3).

**Figure 2:**
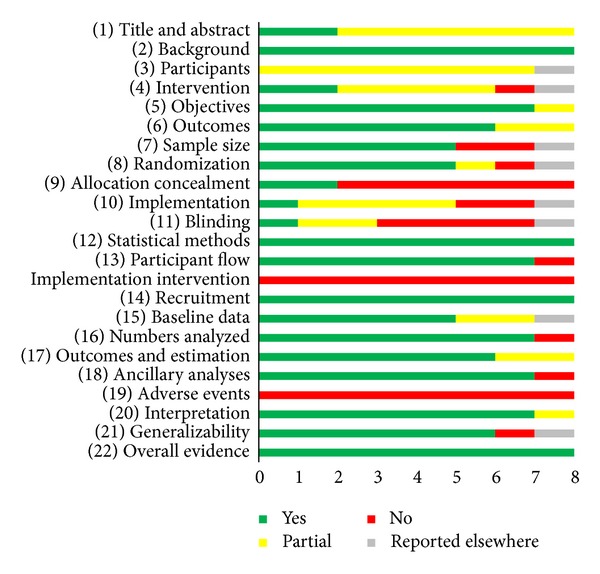
CONSORT checklist of items for reporting trials of nonpharmacologic treatments.

Assessment of the studies using the CONSORT checklist of items for reporting trials of nonpharmacologic treatment also highlighted strengths and weaknesses (Figure 2). The majority of studies included adequately reported background information, study objectives, sample size determination, randomization method, statistical analysis methods, participant flow, recruitment dates, baseline data, numbers analyzed, outcomes, additional analyses, interpretations, generalizability, and overall evidence. Partially complete information was reported in most titles and abstracts. There was limited reporting of the inclusion criteria for study sites and intervention providers as well as the location of data collection. Additionally, only two studies completely described standardization of the intervention and assessment of adherence to the protocol. None of the studies reported adverse event data or results of how the interventions were implemented. As previously stated, the details of allocation concealment were often incomplete or absent, as well as information about blinding of participants and personnel. Of the eight studies, four reported registration in an open access clinical trial registry.

Overall, the studies included had deficiencies in reporting and significant risk of influence of bias.

### 3.6. Meta-Analysis

Six studies reported IBS severity at end of intervention data that was amenable to calculation of effect size; five studies contained data available for pooling for each of IBS severity at postintervention follow-up and quality of life.

Mild-moderate heterogeneity existed between studies with respect to effects of MBT on IBS severity at end of intervention (*I* ^2^ = 49.9%, 95% CI = 0% to 78.2%; Cochran *Q* = 9.982 *P* = 0.076), on IBS severity at postintervention follow-up (*I* ^2^ = 23.3%, 95% CI = 0% to 71.8%; Cochran *Q* = 5.216 *P* = 0.266), and on QOL (*I* ^2^ = 30.4%, 95% CI = 0% to 74%; Cochran *Q* = 5.747 *P* = 0.219).

**Figure 3:**
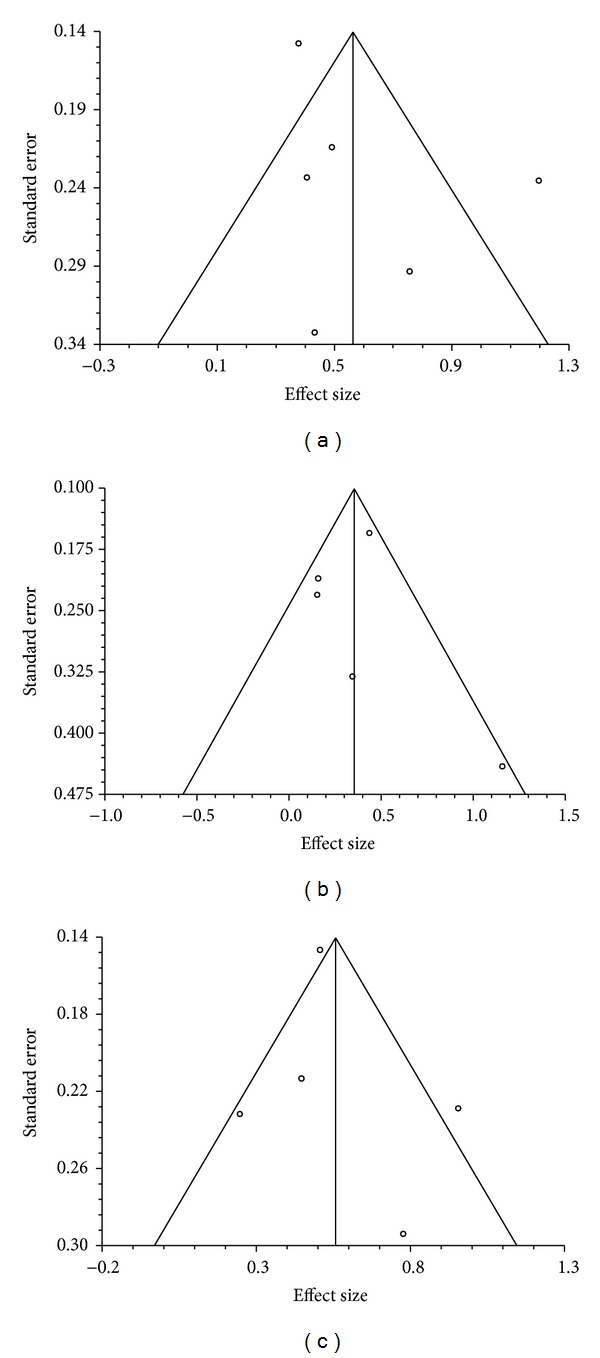
Funnel plots for IBS severity at end of intervention (a), IBS severity at postintervention follow-up (b), and quality of life (c).

Funnel plots (Figure 3), Kendall's tau, and Egger's test for bias are suggestive of low power, low likelihood for unpublished or unreported studies, and not statistically significant for bias across IBS severity at end-of-intervention, (Kendall's tau = 0.333 *P* = 0.469; Egger = 1.901, 95% CI = −4.376 to 8.182 *P* = 0.448), on IBS severity at postintervention follow-up (Kendall's tau = 0.4 *P* = 0.483; Egger = 1.256, 95% CI = −3.988 to 6.501, *P* = 0.501), and on QOL (Kendall's tau = 0 *P* = 0.817; Egger = 1.345, 95% CI = −6.742 to 9.432, *P* = 0.633).

**Figure 4:**
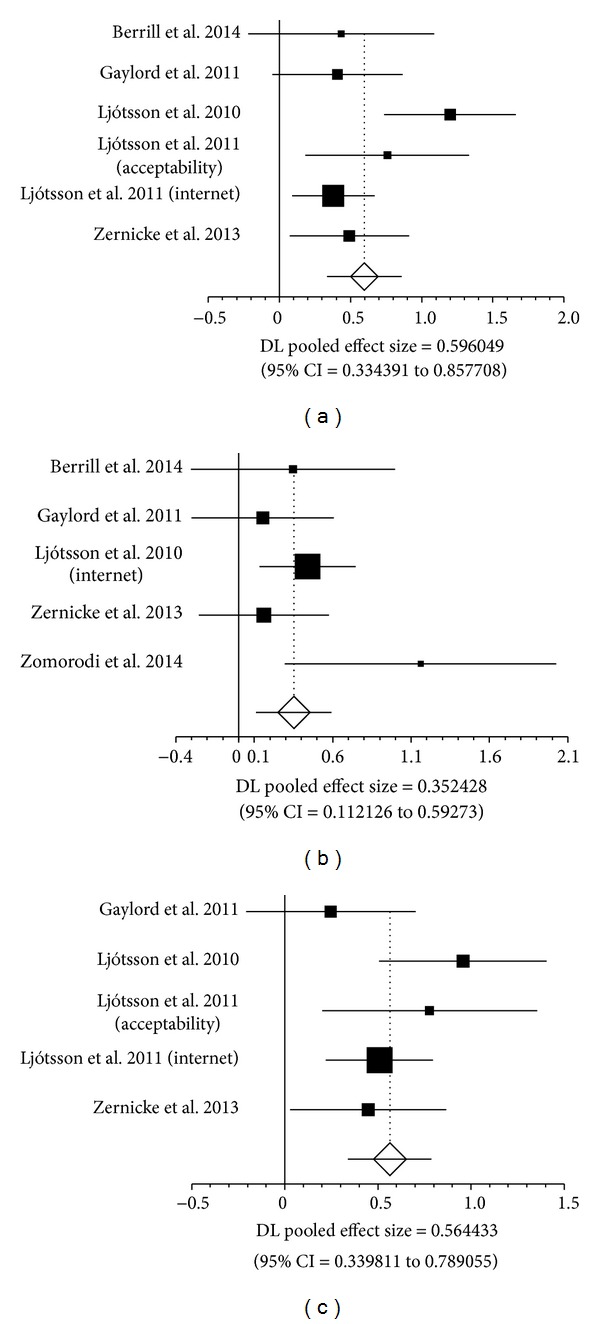
Forest plots for effect size on IBS Severity at end of intervention (a), IBS severity at postintervention follow-up (b), and quality of life (c).

Forest plots (Figure 4) outline a statistically significant pooled effect size for IBS severity at end of intervention (Pooled *d* = 0.596, 95% CI = 0.334 to 0.858), on IBS severity at postintervention follow-up (Pooled *d* = 0.352, 95% CI = 0.112 to 0.593), and on QOL (Pooled *d* = 0.564, 95% CI = 0.340 to 0.789) using random effects model. No major difference in findings was observed using a fixed effects model for pooling data (data not reported).

## 4. Discussion

The results of the studies reviewed suggest that MBT may be an effective treatment for FGIDs achieving both a reduction of symptom severity and an improvement in quality of life. The mean decrease in symptom severity ranged from 23 to 42%. Though the sample size is small, this suggests some consistency in effectiveness observed amongst studies. A previous meta-analysis suggests that the variability of effectiveness of mindfulness therapies is no greater than that observed in other pharmacological or cognitive behavioural therapies across disorders [24]. In Zernicke et al. [20], the mean decrease of 30.7% amongst completers equated to 50% of participants achieving a clinically meaningful reduction in their IBS symptoms (i.e., a reduction of 50 points on the IBS Severity Scale).

### 4.1. Duration of Effect

Additionally, the results suggest that the improvement achieved during treatment is lasting and may even lead to continued improvement. All of the studies that yielded statistically significant improvement in symptom severity at end-of-intervention demonstrated maintenance of that improvement at follow-up. In addition, three studies observed statistically significant improvement in quality of life between end-of-intervention and follow-up. Lasting effects have been observed in previous studies using MBT. One study, which sought to investigate the long-term effects of MBCT in the treatment of depression, found that improvements achieved during treatment were maintained for up to 59.8 months of follow-up [25]. The lasting effects of MBT are likely related to changes in the way participants attend to moment-by-moment cognition, emotion, perception, and sensations—the development of trait or dispositional mindfulness [6].

### 4.2. Quality

Quality assessment of the studies revealed some strengths, but largely weaknesses and deficiencies. Overall, the current literature has not responded to challenges relating to increased quality in design, conduct, and reporting that may impact credibility in the field of mindfulness or other psychological interventions [26].

Some of the studies used active control groups including support groups, discussion forums, cognitive behavioral therapy, and stress-management training. This allowed for participant blinding as well as insight into the mechanism of the effect. In all cases the mindfulness based therapy showed superior efficacy to the other interventions suggesting that the therapeutic benefit is specific to the material covered rather than nonspecific factors such as peer-support, attention, or the expectation effect. However, a major challenge in the study of psychological interventions is the inability to blind all study personnel to participant allocation. Some studies took steps to help conceal allocation and preserve blinding amongst outcome assessors; however no studies took into account blinding of the individuals facilitating the interventions or other steps that might help manage expectation and performance bias.

Another area that posed a risk of bias is incomplete outcome data due to dropouts. MBT requires a large amount of participant involvement and time, often including weekly group sessions and daily home practice. This may have contributed to the high dropout rates observed. Many studies utilized intention to treat analysis to account for these occurrences however some articles did not address this or report the specific manner in which intention to treat analysis was done.

A major limitation to this review is a relatively small number of studies with (qualitatively) significant heterogeneity in their methodology. The follow-up time period varied from two to 18 months. Additionally, the type of intervention varied. Of the seven studies reviewed, three were conducted by the same research group using a unique methodology called internet-based cognitive behaviour therapy (ICBT) which includes mindfulness and acceptance-based exercises in combination with exposure. While it is accessible over the internet, it is not available to the public at this time. In contrast, MBSR and MBCT programs are offered in hospitals, universities and health clinics worldwide.

Most of the studies reviewed enrolled patients with a diagnosis of IBS. The one study that included participants with IBD in remission and IBS-like symptoms was the only study that failed to yield a statistically significant improvement in IBS symptoms. The patients with IBS-type symptoms in this study were a subset of a larger patient population and as a result there was a small sample size which may have contributed to the failure to reach statistical significance. Alternatively, it may be that patients without organic gastrointestinal disease are more responsive to MBT.

Many of the studies had a high percentage of female participants. While there is a risk that this may limit the generalizability of the results it is known that IBS is more prevalent among women [7].

The studies reviewed demonstrated benefits in the placebo groups; however, this is a common finding among trials involving patients with IBS and other subjective complaints. A meta-analysis of the placebo effect in IBS found a range of 16–71% improvement (27) and a randomized controlled trial using open-label placebo for the treatment of IBS demonstrated a statistically significant benefit (28).

Although a statistically significant finding was demonstrated on pooled effect sizes, the low power, small number of studies, and overall high risk of bias in study design or completeness of reporting suggest that this should be interpreted with some discretion.

## 5. Conclusions

Analysis of these studies suggests that mindfulness-based interventions may be useful in improving FGID symptom severity and quality of life with lasting effects; however, substantial improvements in methodological quality must be implemented in future studies in order to fully assess its impact. Due to absence of reporting of adverse events, no definitive conclusions can be drawn with respect to safety. Future studies would benefit from use of established criteria for reporting clinical trials using nonpharmacological interventions, registration of studies in an open-access clinical trial registry, and improvements in blinding to decrease the risk of bias.

## Figures and Tables

**Table 1:** Characteristics and outcomes of studies included in systematic review.

Study	*N*, % female	Population	Intervention & duration	Control	Follow-up	IBS severity at end-of-intervention	IBS severity at follow-up	Quality of life
Berrill et al. 2014 [14]	38, 77%	IBD with IBS-type symptoms	MCT; 16 weeks	Waiting list (TAU)	8 and 12 months	Decrease in IBS-SS but did not reach statistical significance (32.5% vs. 6.8% reduction, *P* = 0.219)	Decrease in IBS-SS but did not reach statistical significance (30.0% vs. 0% reduction, *P* = 0.213)	Not assessed

Gaylord et al. 2011 [15]	75, 100%	IBS	Mindfulness-based stress and pain management program; 8 weeks	Support group	3 months	Significantly greater improvement in IBS-SS (26.4 vs. 6.2% reduction, *P* = 0.006)	Improvement maintained (38.2 vs. 11.8% reduction, *P* = 0.001)	Significant improvement in IBS-QOL at follow-up only (*P* = 0.027)

Lj$\overset{´}{\text{o}}$tsson et al. 2010 [16]	85, 85%	IBS	ICBT, 10 weeks	Online closed discussion forum	3 months	Significant improvement in diary symptom ratings (pain, diarrhea, constipation, and bloating) and GSRS-IBS (42% reduction vs. 12% increase, *P* < 0.001)	Improvement in GSRS-IBS maintained	Significant improvement in IBS-QOL post treatment (*P* = 0.001); further significant improvement at follow-up (*P* = 0.04)

Lj$\overset{´}{\text{o}}$tsson et al. 2011 [17]	Long term follow-up of Lj$\overset{´}{\text{o}}$tsson et al. (2010) [16]				15–18 (mean = 16.4) months		Improvement in GSRS-IBS maintained (*P* < 0.05)	Significant improvement in IBS-QOL (*P* < 0.05), maintained at follow-up; no difference between those who did/did not seek additional care for IBS

Lj$\overset{´}{\text{o}}$tsson et al. 2011 [18]	61, 74%	IBS	ICBT, 10 weeks	Online closed discussion forum before crossing over	12 months	Significantly larger improvement in GSRS-IBS (30.5% reduction vs. 2.8% increase) (Cohen's *d* 0.77 (0.19–1.34 95% CI))	Improvement in GSRS-IBS maintained	Significantly greater improvement in IBS-QOL (Cohen's *d* 0.79 (0.20–1.35 95% CI)); further improvement at follow-up (*P* = 0.04)

Lj$\overset{´}{\text{o}}$tsson et al. 2011 [19]	195, 79%	IBS	ICBT, 10 weeks	Internet-based stress management	6 months	Significantly larger improvement in GSRS-IBS (23.6% vs. 13.1% reduction) (difference in score of 4.8 (1.2–8.4 95% CI))	Significantly larger improvement in GSRS-IBS (difference in score of 5.9 (1.9–9.9 95% CI)); nonsignificant trend towards continued improvement	Significantly larger improvement in IBS-QOL (difference in score of 10 (4.5–15.6 95% CI)), maintained at follow-up (difference in score of 6.2 (0.2–12.2 95% CI))

Zernicke et al. 2013 [20]	90, 90%	IBS	MBSR; 8 weeks	TAU waitlist	6 months	Significantly greater improvement in IBS-SS (30.7 vs. 5.2% reduction *P* < 0.0001 among completers, 16.9% vs. 3.5% using ITT)	Improvement maintained; some improvement seen in TAU group leading to no statistically significant difference (*P* = 0.17)	IBS-QOL improved in both groups posttreatment and follow-up (*P* < 0.001)

Zomorodi et al. 2014 [21]	48, 44%	IBS and healthy controls	MBSR or CBT, 8 weeks	No psychological intervention	2 months	Not provided	Significantly greater improvement in IBS questionnaire vs. CBT or control (35.0% vs. 5.8%, *P* < 0.05)	Not assessed

GSRS-IBS: gastrointestinal symptom rating scale—IBS version.

ICBT: internet-based cognitive behavior therapy which includes exposure, mindfulness, and acceptance.

IBS-SS: irritable bowel syndrome severity score.

IBDQ: inflammatory bowel disease questionnaire.

IBS-QOL: irritable bowel syndrome quality of life instrument.

MCT: multiconvergent therapy-combination of mindfulness meditation and CBT.

MBSR: mindfulness-based stress reduction.

TAU: treatment as usual.

**Table 2:** Cochrane risk of bias assessment of studies included in systematic review.

Reference	Random sequence generation (selection bias)	Allocation concealment (selection bias)	Blinding of participants and personnel (performance bias)	Blinding of outcome assessment (detection bias)	Incomplete outcome data (attrition bias)	Selective reporting (reporting bias)	Other bias	Overall
Berrill et al. 2014 [14]	Low	Low	High	Unclear	High	Low	Low	High
Gaylord et al. 2011 [15]	Low	Unclear	Low∗	Low	Unclear	Low	Low	Unclear
Lj$\overset{´}{\text{o}}$tsson et al. 2010 [16]	Low	Low	High	Unclear	Low	Low	Unclear	High
Lj$\overset{´}{\text{o}}$tsson et al. 2011 (long term) [17]	As Lj$\overset{´}{\text{o}}$tsson et al. 2010 [16]	As Lj$\overset{´}{\text{o}}$tsson et al. 2010 [16]	As Lj$\overset{´}{\text{o}}$tsson et al. 2010 [16]	As Lj$\overset{´}{\text{o}}$tsson et al. 2010 [16]	Low	Low	Low	High
Lj$\overset{´}{\text{o}}$tsson et al. 2011 (Acceptability) [18]	Low	Low	High	Unclear	Unclear	Low	Low	High
Lj$\overset{´}{\text{o}}$tsson et al. 2011 (Internet) [19]	Low	Low	Low∗	Unclear	Low	Low	Low	Unclear
Zernicke et al. 2013 [20]	Low	Unclear	High	Unclear	Unclear	Low	Low	High
Zomorodi et al. 2014 [21]	Unclear	Unclear	Low∗	Unclear	Unclear	High	Unclear	High

Low∗: study participants were blind; however due to the nature of a psychological intervention, those providing the intervention were not blind.
